# Resolution-Free Accurate DNA Contour Length Estimation from Atomic Force Microscopy Images

**DOI:** 10.1155/2019/4235865

**Published:** 2019-06-09

**Authors:** Peter I. Chang, Ming-Chi Hsaio

**Affiliations:** Mechanical Engineering, National Taiwan University of Science and Technology, Taiwan

## Abstract

This research presented an accurate and efficient contour length estimation method developed for DNA digital curves acquired from Atomic Force Microscopy (AFM) images. This automation method is calibrated against different AFM resolutions and ideal to be extended to all different kinds of biopolymer samples, encompassing all different sample stiffnesses. The methodology considers the digital curve local geometric relationship, as these digital shape segments and pixel connections represent the actual morphology of the biopolymer sample as it is being imaged from the AFM scanning. In order to incorporate the true local geometry relationship that is embedded in the continuous form of the original sample, one needs to find this geometry counterpart in the digitized image. This counterpart is realized by taking the skeleton backbone of the sample contour and by using these digitized pixels' connection relationship to find its local shape representation. In this research, one uses the 8-connect Freeman Chain Code (CC) to describe the directional connection between DNA image pixels, in order to account for the local shapes of four connected pixels. The result is a novel shape number (SN) system derived from CC, which is a fully automated algorithm that can be applied to DNA samples of any length for accurate estimation, with efficient computational cost. This shape-wise consideration is weighted to modify the local length with great precision, accounting for all the different morphologies of the biopolymer sample, and resulted with accurate length estimation, as the error falls below 0.07%, an order of magnitude improvement compared to previous findings.

## 1. Introduction

The Atomic Force Microscopy (AFM) system has the ability to probe samples at the nanometer scale, owing to its ability in sensing the sample surface to resolve force interaction at the pico-Newton level [1]. This feature makes AFM systems a useful imaging device in the field of nanotechnology, molecular biology, and many others. It is well known in AFM's biological application to image biopolymers thanks to its ability to image in liquid, the biopolymer's natural environment [2].

**Figure 1:**
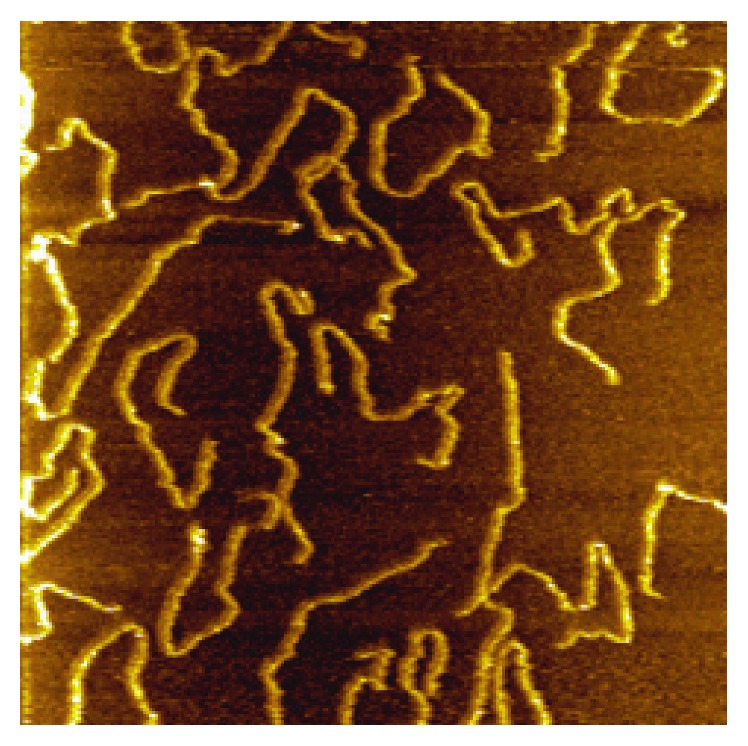
DNA image acquired by the atomic force microscopy system [4].

One very interesting characteristic is the length of a single DNA strand, denoted as *l*_*c*_. This contour length can be applied to identify genome editing results and other application outcomes [3]. And accuracy in getting *l*_*c*_ correct is essential at this scale, as there is small room for error in genome editing, since one base-pair distance for DNA is only 0.34 nm. Thus, AFM images of DNA samples provide means for such *l*_*c*_ studies on accurate DNA length estimation, like the image illustrated here in Figure 1.

**Figure 2:**
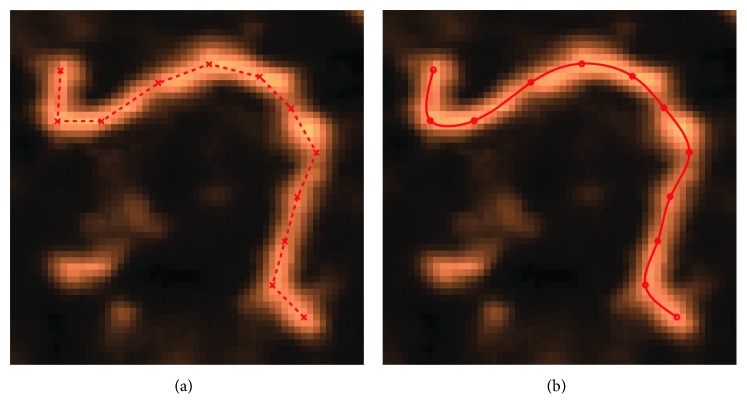
(a) Linear/straight fitting with selected points manually. (b) Cubic spline fit utilizing the same manual selective points on the AFM image.

There are two ways in finding *l*_*c*_ from AFM images. One is by manual fitting, and the other is by automatic skeleton tracing with image processing. Fitting typically relies on human operators picking specific positions along the DNA contour by examination on the acquired image, which relies on the trained eye of a scholar to map out the contour length *l*_*c*_ [5, 6], as illustrated in Figure 2.

**Figure 3:**
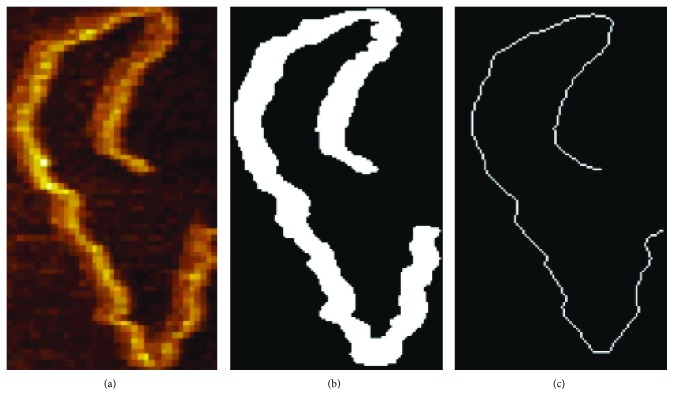
Illustration of DNA image preprocess to extract contour skeleton backbone. (a) Original AFM image cut-out with a single DNA sample. (b) Binary image acquired by thresholding the original. (c) Skeleton extracted by a repetitive thinning process to produce a single-pixel width, connected contour.

On the other hand, the automatic *l*_*c*_ estimation traces the DNA image along its backbone skeleton. This is done by thinning the strand image to its median position from the overall acquired outline and retaining only the skeleton of the DNA, as is illustrated in Figure 3.

**Figure 4:**
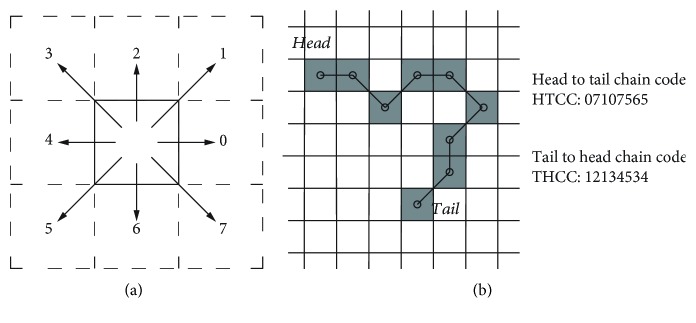
(a) 8-connect chain code connectivity from the center pixel, illustrating the connection code 0∼7 associated according to the adjacent pixel location. (b) An example of a single-pixel width biopolymer skeleton backbone used for deriving the chain code. Starting from head to tail codes: 07107565 and tail to head codes: 12134534.

The backbone extracted from the original AFM image is a *single-width* connected pixel arranged with the following rule: only one adjacent pixel is allowed to connect to the central pixel to form a continuous contour, either directly (horizontal or vertical) or diagonally, as is illustrated in Figure 4.

From this single width contour pixel arrangement, a continuous *chain code* (CC), defined as *C* = *c*_1_*c*_2_ ⋯ *c*_*n*_, can be formed by tracing the connectedness of adjacent pixels from the skeleton's one end to the other, according to the 8-connect Freeman's eight directions [7].

Researchers have been using the Freeman CC to estimate *l*_*c*_, by counting the number of even and odd occurrences along the DNA skeleton, which is to trace along the chain code, *C* = *c*_*i*_, *i* = 1 ~ *n*, and tally up the occurrences of even number chain codes (*n*_e_) as well as its odd occurrences (*n*_o_).

Since the even chain code connects adjacent pixels directly (vertically/horizontally), and the odd CC connects diagonally, one estimates *l*_*c*_ first by finding the Euclidean length (norm) of all the pixel center connections and then multiply the pixel resolution *r* to find *l*_*c*_. This is defined as the Freeman estimator $L_{\text{F}}=r\left(n_{\text{e}}+\sqrt{2n_{\text{o}}}\right).$ [8].

However, *L*_F_ lacks the accuracy that is required in these microscopy systems. Thus, there are researches that made modifications to *L*_F_. These include the Kupla estimator (*L*_K_) and the corner estimator *L*_C_. *L*_K_ modified the diagonal √2 values due to digital slope inclination, and *L*_C_ further accounts for tight turns geometrically. Thus, in the end *L*_K_ and *L*_C_ end up with different coefficients from *L*_F_ [9].

There were further researches to improve *l*_*c*_ accuracy. One research smooths out the digitized pixilation of the contour skeleton backbone and applied a spatial Fourier transform on the image. Through tuning the Gaussian filter in 2D, a smother *l*_*c*_ is estimated [10].

Other than modifying the pixel connection Euclidian length, another research modifies *l*_*c*_ by adjusting the pixel center coordinate representation *x*_p_. A weight *k* is added to modify the coordinate location by considering the three consecutive points with *X*_p_ = *k*(*x*_p−1_ − *x*_p_) + *x*_p_ + *k*(*x*_p+1_ − *x*_p_). This length estimator *L*_P_ calculates *l*_*c*_ according to the modified *X*_p_ [11].

Another *l*_*c*_ estimator is designed specifically for DNA strand samples, named *L*_DNA_. This estimator introduced a nominal coefficient for different DNA lengths and is defined as *L*_DNA_ = *rC*_*f*_(*n*_e_+√2*n*_o_), where *C*_*f*_ is inversely calculated from simulated *l*_*c*_ data, so a table of *C*_*f*_ helps *L*_DNA_ to match the expected value of *l*_*c*_ [12].

More recently, a machine learning approach utilized a feature extraction to fit different cubic spline segment occurrences with the following: *horizontal*, *vertical*, *diagonal*, *perpendicular*, variating *height* and *thickness*, as defined by {*n*_horz_, *n*_vert_, *n*_diag_, *n*_perp_, *n*_htcv_, *n*_tkcv_} [13]. This machine learning estimator *L*_ML_ is trained to generate coefficients considering the abovementioned feature from known DNA *l*_*c*_.

A summary table in Table 1 provides a quick review of the abovementioned *l*_*c*_ estimators.

In this paper, the authors propose an estimator based on the DNA imaged contour shape, thus having the name *Shape* estimator *L*_S_, where *L*_S_ is designed to be robust to image resolution and only uses minimal computational resource. This is achieved by considering the neighboring shape of the original two-pixel connection inspired from *L*_F_, but as all the DNA local morphology shapes are considered for estimating *l*_*c*_, the resultant accuracy is shown to improve by more than an order of magnitude.

Detailed methodology of the *L*_S_ estimator is explained in Section 2, starting from the general image preprocessing to the identification of twelve local 4-pixel segment configuration shapes. Then, the 12-shape correction coefficients *k*_1_ ~ *k*_12_ are calibrated in Section 3, with different resolutions considered. Finally, the *l*_*c*_ values for *L*_S_ are compared with *L*_DNA_ and *L*_F_ in Section 4.

## 2. Contour Length Estimation with Local Shape Consideration

*L*_S_ estimation essentially takes into account the local shape considerations. As two neighboring pixels are connected together in this AFM image, the overall shape around the two connected pixels represents different local lengths as this DNA morphology is observed. In a tight turn; i.e., a “kink,” this local length will certainly be longer then a smooth linear local profile.

Thus, *L*_S_ considers the two additional pixels extending from the center two-pixel connection and identifies the different 4-pixel segmented shapes surrounding along the DNA skeleton backbone. Then, *L*_S_ makes shape-corrected length adjustments, by multiplying the local shape's corresponding coefficient to adjust for the estimated *l*_*c*_. It can be observed that the extension of this segmented elemental shape is not limited to 4 pixels, as with more pixels such as a 5-pixel segment can also be considered. However, due to the trade-off for computational cost and performance, this research investigates the *L*_S_ with 4-pixel elements.

### 2.1. Pixel Resolution and Image Preprocessing

**Figure 5:**
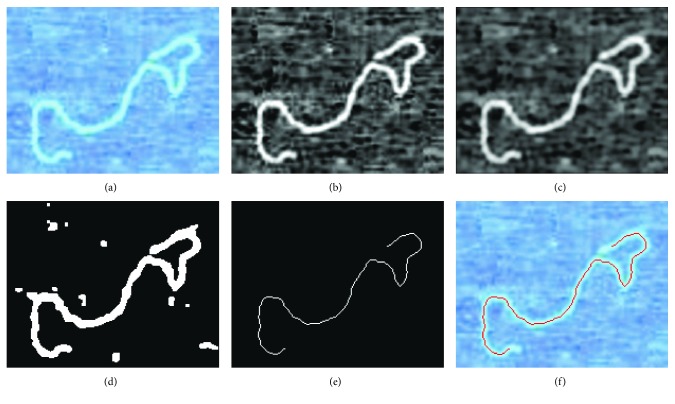
DNA image preprocess. (a) Original image, (b) binary transform, (c) image filtering, (d) image thresholding, (e) thinning and debranching and (f) final result compared with the original image.

A standard preprocess extracts the DNA image into the *l*_*c*_′s skeleton backbone, by thinning the DNA strand into the centerline of the biopolymer. This research's automatic image process is illustrated here in Figure 5.

First, the DNA image is prefiltered and mapped into a binary image with thresholding. Then, further, 2-D filters remove isolated pixel islands, ensuring that a single DNA contour is captured. And finally, an iterative debranch thinning morphology is applied to find the skeleton backbone that can be chain-coded [14].

It is well known that AFM systems have a tip broadening effect when imaging, which expands the DNA strand width to a larger value. A repeated thinning preprocess in average converges the single-width pixel contour, towards the mid-point of the DNA strand automatically, given an AFM image with enough resolution across the DNA width [15].

### 2.2. Identification of 4-Pixel Segment Shape Connectivity

Given the resultant single-width pixels *P* = {*p*_*i*_, *i* = 0 ~ *n*} for the contour's skeleton backbone, its CC (*C* = *c*_*i*_*i* = 1 ~ *n*) is coded from one end to the other. Note that this research utilizes the 8-connect chain code, resulting in integers ranging from 0 ~ 7 for all *c*_*i*_ and that *c*_*i*_ is one off from *p*_*i*_, as there are *n* connections between *n* + 1 pixels.

**Figure 6:**
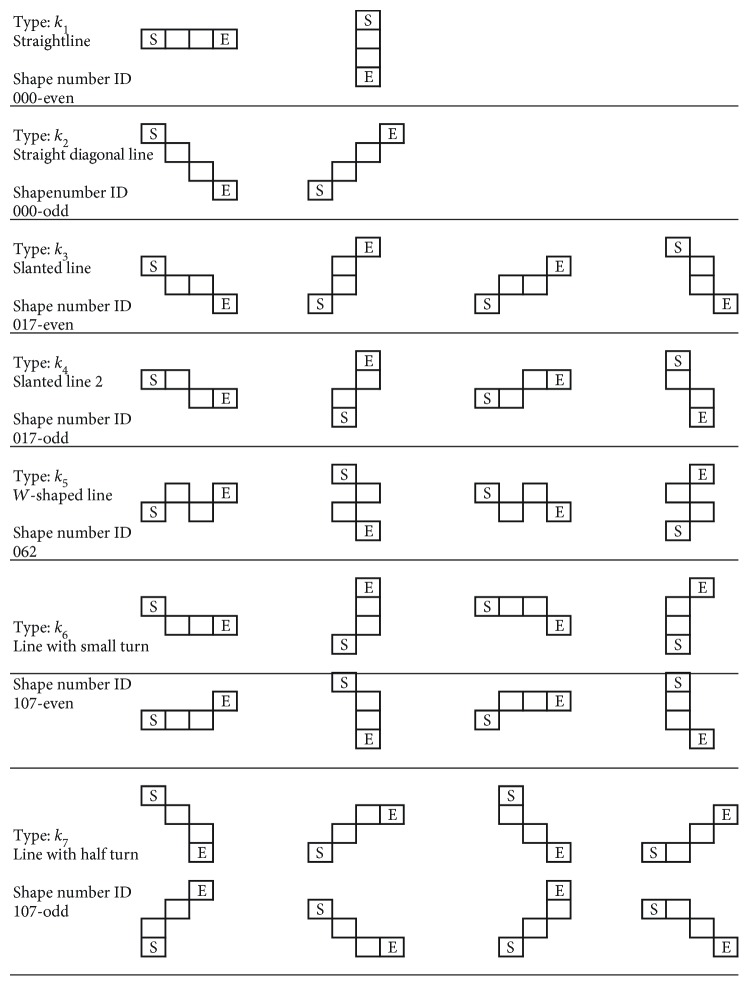
Four-pixel segments of the shapes for *k*_1_ ~ *k*_7_, with their associated rotation and mirror alternatives.

**Figure 7:**
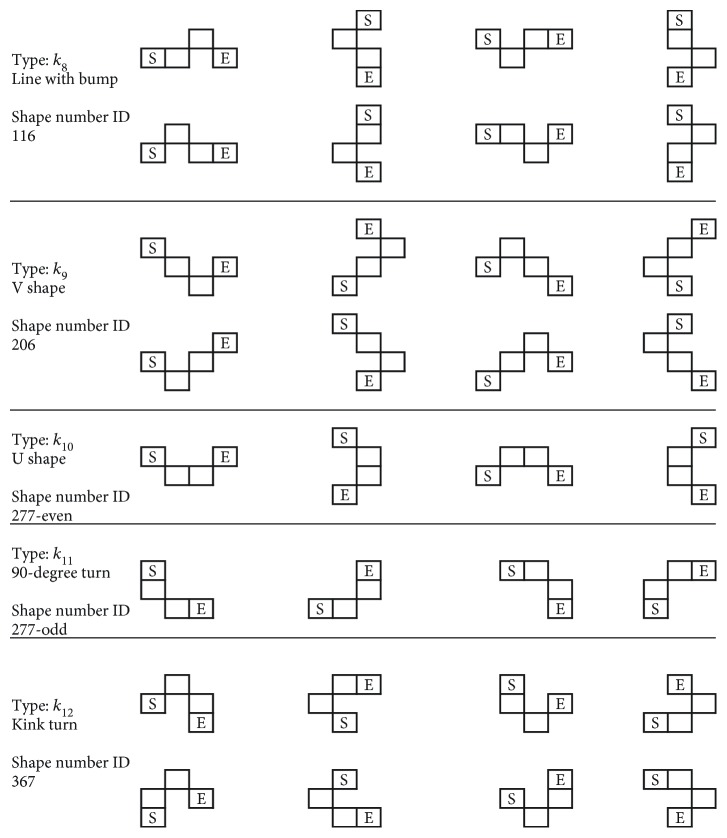
Four-pixel segments of the shapes for *k*_8_ ~ *k*_12_, with their associated rotation and mirror alternatives.

With the 4-pixel segment setup, there are up to a total of 64 ways (4^4^) to connect the 4 pixels into single-pixel width arrangements. This research paper has fully outlined all the possibilities, and the full table of all 64 different single-width 4-pixel segments is arranged in Figures 6 and 7. They are arranged by the assigned *k*_1_ ~ *k*_12_ types, with all the same types grouped together.

**Figure 8:**
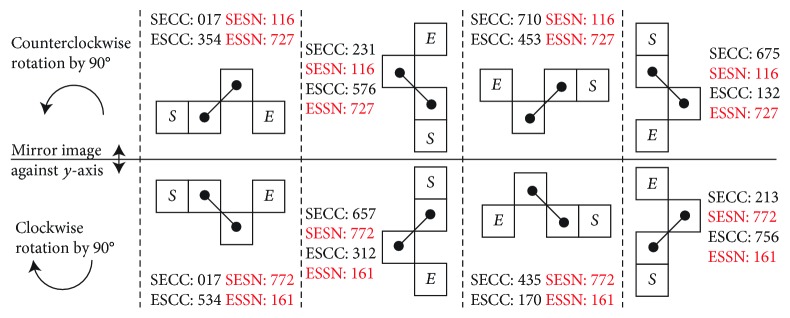
Rotational and mirror representation of *k*_8_ shape 4-pixel segment.

It is clear that all the same types of *k*_*j*_ shape are grouped with the 4-pixel segment's mirror and rotational images. Take for example the *k*_8_ shape, where the segment is rotated clockwise/counterclockwise for 90 degrees individually and mirrored on the *y*-axis, shown here in Figure 8.

**Figure 9:**
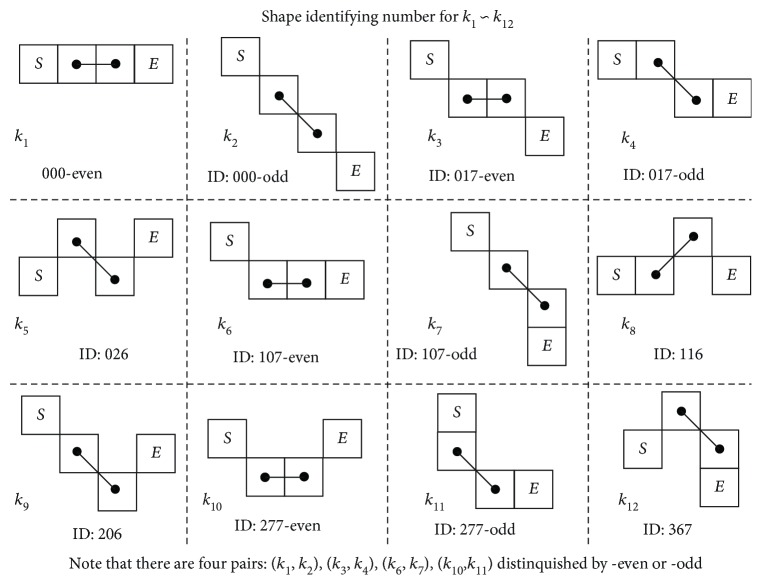
Geometric connectivity of the twelve independent *k*_1_ ~ *k*_12_ shapes for 4-pixel segments. The inner two direct connections (horizontal/vertical and diagonal) are connected with two additional outward pixels, accounting for the local 2D morphology change.

Having these *k*_1_ ~ *k*_7_ segment shapes distinguished, the original inner 2-pixel connection's distance can now be corrected, by considering the outward extended 4-pixel segment shape. This would take into account the local geometric features according to its categorized shape. Since the skeleton backbone is composed of consecutive 4-pixel segments all along its contour, when tracing from one end to the other, this research makes sure that the *L*_S_ estimator identifies every 4-pixel segment to the *twelve* unique *k*_*j*_ shapes, as shown in Figure 9.

#### 2.2.1. Chain Code, Shape Number, and Identifier

In order to identify a skeleton backbone's different 4-pixel segment shapes along the contour, this research utilizes its chain code, formed as a series of integer number, and developed a novel algorithm called the shape number (SN) identification, labeled *S*, and uses it to derive an exclusive identifier (ID) number for matching the abovementioned unique *k*_1_ ~ *k*_12_ shapes.

A typical CC collection, *C* = {*c*_1_*c*_2_*c*_3_ ⋯ *c*_*n*−2_ *c*_*n*−1_*c*_*n*_}, is a series of integers made from 0 ~ 7, provided the *n* + 1single-width skeleton backbone pixels *P* = {*p*_0_*p*_1_*p*_2_ · ·*p*_*n*__−1_*p*_*n*_}. Note that *C* is one-off from *P* and that *p*_*i*_*s* is numbered from 0 ~ *n*. This research emphasizes the general ability to distinguish any skeleton backbone, and while for any given backbone, it creates a set of two distinct CC for every skeleton, due to starting the connection from different ends of the pixel chain. The algorithm will demonstrate the ability to converge on the distinguished 4-pixel segment shapes.

**Figure 10:**
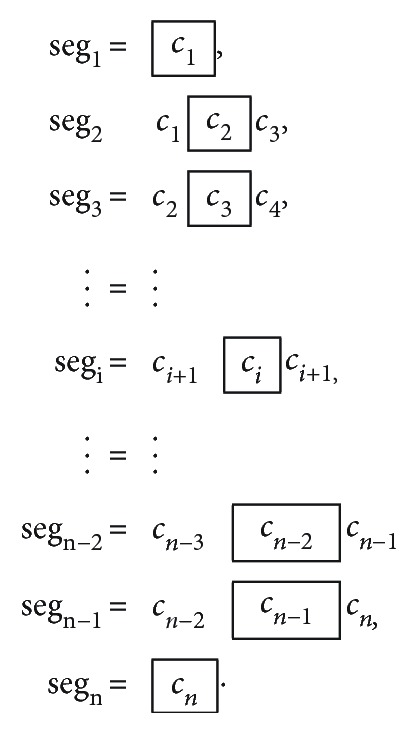


As the algorithm needs to continuously identify the 4-pixel segments throughout the contour backbone, a rolling window starts from any end of *C* and collects the following *n*-segments (Figure 10)

(1) $$\begin{array}{c} \text{Seg}=\left\{\text{se}{\text{g}}_{1},\text{se}{\text{g}}_{2},\cdots ,\text{se}{\text{g}}_{\text{i}},\cdots ,\text{se}{\text{g}}_{n}\right\}. \end{array}$$

**Figure 11:**
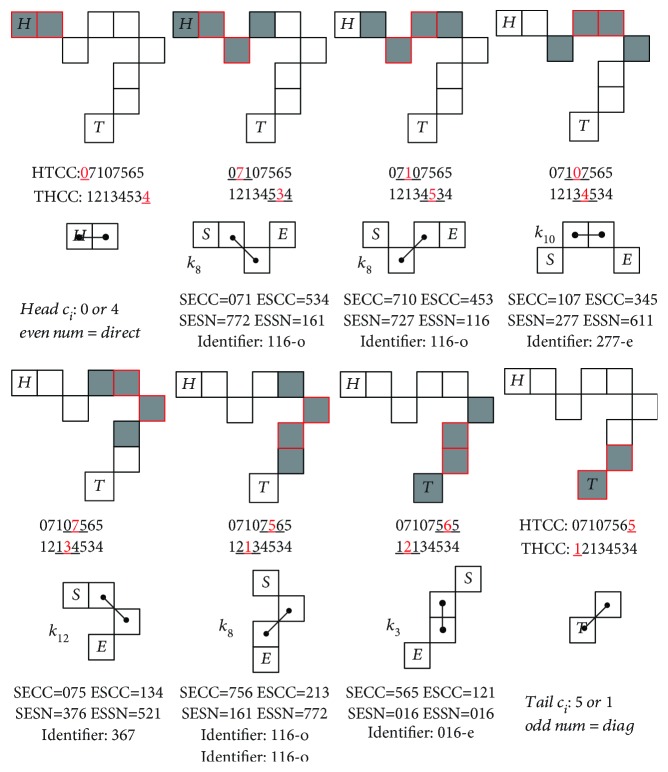
Consecutive determination of the 4-pixel segment shape number through the rolling window. Two sets of chain code provided HTCC (head to tail) and THCC (tail to head), with their associated local segment CC, also bidirectional: SECC (start to end) and ESCC (end to start), which leads to their derived SESN (start to end) and ESSN (end to start).

It is clear that each seg_*i*_ segment is comprised of three consecutive chain codes (*c*_*i*−1_, *c*_*i*_, *c*_*i*+1_), since a 4-pixel segment consists of three connections. With the exception of the first and last segments of the contour skeleton, where there are not enough pixels to form a 4-pixel segment, thus the algorithm just takes the original two connecting pixels, i.e., the original *c*_1_ or *c*_*n*_. The pixel/geometric representation of a rolling window CC segmentation is demonstrated in the Figure 11.

Notice that the rolling window in *C* moves the 4-pixel segment consecutively from *Head* to *Tail*, and each segment can be coded as seg_*i*_ = {*c*_*i*−1_, *c*_*i*_, *c*_*i*+1_}, from *i* = 2 ~ *n* − 1, excluding *Head* (*i* = 1) and *Tail*(*i* = *n*).

This research now defines a shape number (SN), derived from each of the rolling segments as

(2) $$\begin{array}{c} S_{\text{seq}}=\left\{s_{\mathrm{seg}2}s_{\mathrm{seg}3}s_{\mathrm{seg}4}\cdots s_{\mathrm{seg}n-3}s_{\mathrm{seg}n-2}s_{\mathrm{seg}n-1}\right\}. \end{array}$$

In short, *S* = {*s*_seg*i*_, *i* = 2 ~ *n* − 1} is a collection of the ordered cyclic difference from each segment's continuous 3 chain codes. Thus, for each segment, SN is composed of three integer numbers as *S*_seg*i*_ = {*s*_*i*−1_, *s*_*i*_, *s*_*i*+1_}, defined as

(3) $$\begin{array}{c} s_{{\mathrm{seg}}_{i}}=\left\{\begin{array}{c} s_{i-1}=c_{i-1}-c_{i+1},\\ s_{i}=c_{i}-c_{i-1},\\ s_{i+1}=c_{i+1}-c_{i}, \end{array}\right\}. \end{array}$$

Since all CC is comprised of integers from 0 ~ 7, SNs (*s*_*i*_) are also retained between 0 ~ 7. Thus, whenever *s*_*i*_ is derived as negative, we automatically take 8's compliment to correct it, with *s*_*i*_ = *s*_*i*_ + 8 if *s*_*i*_ < 0.

One such example of SN derived is illustrated in the lower part of Figure 11, where the SN is calculated from both directions of the chain code inside each segment: the *Start to End Chain Code* (SECC) as well as the *End to Start Chain Code* (ESCC). It is obvious that SECC and ESCC are different; therefore, the resulting *Start to End Shape Number* (SESN) and *End to Start Shape Number* (ESSN) are also derived different, albeit representing the exact same segment.

To ensure exclusive identification on the same 4-pixel segment, for both bidirectional CC and SN coding, in addition to all the same shape mirroring and rotational configuration, a simple unique identifier (ID) number is needed to match the rolling 4-pixel segments to the *k*_1_ ~ *k*_12_ shapes.

#### 2.2.2. Unique Identifier (ID) Matching

In order to deal with such bidirectional, mirroring, and rotational segment ambiguity, the following rule has been applied to ensure a single SN identifier (ID) to match explicitly one *k*_1_ ~ *k*_12_ shape, for any given random shape number in the lengthy *l*_*c*_ contour skeleton backbone.

This is provided by examining all the SESN and ESSN for all the *k*_1_ ~ *k*_12_ shapes and capturing the combinatory relative adjacent arrangements from the 4-pixel segment geometry, i.e., reordering the representative numerals of SN to allow for the direct/diagonal connections, to make representation of the given shape.

Since the rolling window segmental SN *s*_seg*i*_ will fall into the recognizable *k*_1_ ~ *k*_12_ shapes, the ID number can be derived from the known segment numbers as specified in Figure 9. Thus, the identifier is a unique number for each of the shape *k*_*j*_, *j* = 1 ~ 12, such that when the rolling window covers a 4-pixel segment, by performing this numeral operation (algorithm), one will find the identifier.

The following Algorithm (1) outlines the unique identifier (ID)'s reorder methodology for all of the *k*_1_ ~ *k*_12_ shapes.

The rules for the identifier are stated as follows:

*Unique*—there exists one unique ID number for each of *k*_5_, *k*_8_, *k*_9_, and *k*_12_ shapes.

*Common*—there exists *common* ID numbers for the following pairs: {*k*_1_, *k*_2_}, {*k*_3_, *k*_4_}, {*k*_6_, *k*_7_}, and {*k*_10_, *k*_11_}.

*Distinguish* **—**the aforementioned sets are discerned by the connection type of the center pixels, {direct or diagonal}, by checking its original CC number *c*_*i*_, with even/odd numbers representing direct/diagonal, respectively.

Take for example the *k*_8_SN, as there exist four different SN combinations: 116, 772, 727, and 161 form shaping of the eight different segments, as shown in the last row of Figure 8, due to coding of all the different mirrors/rotations and bidirectional CC.

After performing the abovementioned ID algorithm, we are able to uniquely transform all SN to the same identifier (ID) number: 116, as shown from Table 2.

Finally, the algorithm arrives with *k*_5_, *k*_8_, *k*_9_, and *k*_12_ matching up with their respective ID numbers: 026, 116, 206, and 367. In addition, the algorithm matches the pairs {*k*_1_, *k*_2_}, {*k*_3_, *k*_4_}, {*k*_6_, *k*_7_}, and {*k*_10_, *k*_11_} commonly to 000, 017, 107, and 277, respectively. In order to distinguish between the pairs, the original {direct, diagonal} connection is once again used: by checking if the original *c*_*i*_ is either *even* or *odd*, then it can be trivially matched to the correct shape in the pair Table 3.

All the ID numbers are listed for *k*_1_ ~ *k*_12_ shapes here in Figure 9, derived from all the 64 shapes in Figures 6 and 7. Note that the common ID numbers are annotated with (-even/-odd) for distinguishing.

### 2.3. *k*_*j*_ Parameter Equation Representation

Now that the unique ID number is obtained, it is then ready to amass a collection of the different samples of a given *l*_*c*_ length, in order to retrieve the correction parameter for the different *k*_*j*_s, provided with the same DNA characteristics, i.e., with a fixed *l*_*p*_ = 50 nm.

#### 2.3.1. Length Calculation with Coefficients

This is first done by identification on one individual DNA sample's contour, by summing up each shape component *k*_*j*_′s occurrence contribution for its segment's connection length. In other words, one identifies along the skeleton backbone and tallies the individual occurrences of the twelve *k*_*j*_s, multiplied by the corresponding connection length (1 or √2 pixel length) along with its correction coefficient. This makes the sum of all the length contributions equal to the contour length *l*_*c*_ as

(4) $$\begin{array}{c} l_{c}=r\left[\overset{12}{\underset{j=1}{\sum }}n_{kj}k_{jl}k_{j}+\left(l_{\text{H}}+l_{\text{T}}\right)\right], \end{array}$$

where *n*_*kj*_, *j* = 1 ~ 12 is the number of occurrences of the type *k*_*j*_ shapes, provided from the identification along the skeleton backbone. *k*_*j*_ is the correction coefficient, and *l*_*kj*_ is the connection length (either 1 or √2 according to the *k*_*j*_ shape). *l*_H_ and *l*_T_ are the head and tail length, respectively, and finally *r* is the AFM image pixel resolution.

From Figure 9, *l*_*k*_1__ ~ *l*_*k*_12__ length is ordered in Table 4.

#### 2.3.2. Matrix Form for Inverse Calculation

The second step is to collect a sufficient amount of representation of this same type of biopolymer samples and list all the length equations based on these samples. The logic is that with multiple samples of the same kind, imaged under the same pixel resolution, the *k*_*j*_ shapes collectively represent the same type of twist/turn, resulting in the same length contribution for the same class of biopolymers *l*_*c*_.

Combining equation (4) with the associated *l*_*kj*_ values in Table 4, we arrive at

(5) $$\begin{array}{c} lm,c=r\left[\left(n_{m,k_{1}}k_{1}\left(1\right)+n_{m,k_{2}}k_{2}\left(\surd 2\right)+n_{m,k_{3}}k_{3}\left(1\right)+n_{m,k_{4}}k_{4}\left(\surd 2\right)+n_{m,k_{5}}k_{5}\left(\surd 2\right)+n_{m,k_{6}}k_{6}\left(1\right)+n_{m,k_{7}}k_{7}\left(\surd 2\right)+n_{m,k_{8}}k_{8}\left(\surd 2\right)+n_{m,k_{9}}k_{9}\left(\surd 2\right)+n_{m,k_{10}}k_{10}\left(1\right)+n_{m,k_{11}}k_{11}\left(\surd 2\right)+n_{m,k_{12}}k_{12}\left(\surd 2\right)\right)+\left(l_{m,\text{H}}+l_{m,\text{T}}\right)\right], \end{array}$$

for any given backbone skeleton length *l*_*m*,*c*_, given the index *m*'th sample. Equation (5) can be represented with a matrix form, with

(6) $$\begin{array}{c} r\left(NDK+B\right)=L, \end{array}$$

where

(7) $$\begin{array}{c} N=\left[\begin{array}{cccc} n_{1,k_{1}} & n_{2,k_{2}} & \cdots & n_{1,k_{12}}\\ n_{2,k_{1}} & n_{2,k_{2}} & \cdots & n_{2,k_{12}}\\ \vdots & \vdots & \ddots & \vdots \\ n_{m,k_{1}} & n_{m,k_{2}} & \cdots & n_{m,k_{12}} \end{array}\right],\\ K=\left[\begin{array}{c} k_{1}\\ k_{2}\\ \vdots \\ k_{12} \end{array}\right], \end{array}$$

and

(8) $$\begin{array}{c} B=\left[\begin{array}{c} b_{1}\\ b_{2}\\ \vdots \\ b_{m} \end{array}\right], \end{array}$$

and

(9) $$\begin{array}{c} L=\left[\begin{array}{c} l_{c,1}\\ l_{c,2}\\ \vdots \\ l_{c,m} \end{array}\right] \end{array}$$

provided *D* = diag(1, √2, 1, √2, √2, 1, √2, √2, √2, 1, √2, √2) is a 12-by-12 square matrix, such that DK = [*k*_1_,√2*k*_2_, *k*_3_,√2*k*_4_,√2*k*_5_, *k*_6_,√2*k*_7_,√2*k*_8_,√2*k*_9_, *k*_10_,√2*k*_11_,√2*k*_12_]^T^.

Note that *b*_*m*_ = *l*_h,*m*_ + *l*_t,*m*_ is the *head* and *tail* (boundary) connection length summation. Also note that *N* is an *m-*by-12 matrix, *K* is 12 by 1, and *B* and *L* are both *m-*by-1 matrices.

The final procedure here is to derive matrix *K* using a standard linear regression and find the best fit for the *k*_1_ ~ *k*_12_ value. The final results are presented in the next section.

## 3. Contour Parameter Calibration Result

In order to guarantee convergences of the *k*_1_ ~ *k*_12_ coefficients, different known values of *l*_*c*_ and *r* single-pixel-width AFM images were simulated for *k*_*j*_ calibration. Due to the combinations of different *l*_*c*_ and *r*, plus a surplus amount of samples for each (*l*_*c*_, *r*) pair, a total of 58,800,000 images were generated.

All the simulated images are based on DNA characteristics, as mentioned in Introduction, where all the samples have the same persistance length of *l*_*p*_ = 50 nm.

The different lengths calculated ranged from 340 to 1020 nm, for every 34 nm, and the different resolution *r* is simulated between 5.1 and 7.8 nm/pixel, with a 0.1 nm interval. Thus, there are 21 different *l*_*c*_ scales, along with 28 altering *r*, making a total of 21 × 28 = 588 test cases. Each case is studied with 10,000 DNA images, for sufficient representation on *k*_*j*_s. In order words, the test index *m* = 10,000 was used for equation (6).

### 3.1. Convergence of the *k*_*j*_ Coefficient

**Figure 12:**
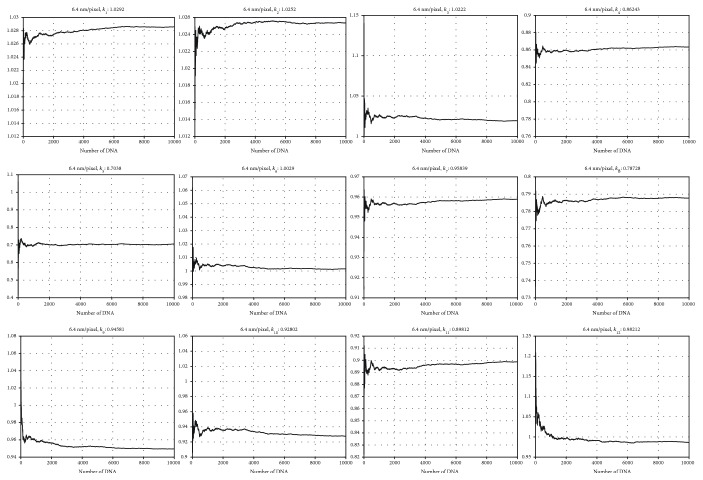
Convergence of all *k*_1_ ~ *k*_12_ coefficients, with growing number of *m* = 2.1 × 10^6^ image samples. Resolution at 6.4 nm/pixel, midrange from all simulation data.

This research first checks the convergence of all *k*_*j*_ coefficients, given a growing number of image files, i.e., growing number of *m* in equation (6). The results are illustrated in Figure 12.

All coefficients verify its convergence when given more than 0.5 million samples and remain constant with fluctuation of less than 0.01% after 1 million samples. This result is verified for all resolutions *r* = 5.1 ∼ 7.6 nm/pixel, showing similar trend for all *k*_*j*_s.

### 3.2. Linear Variation of *k*_*j*_ Dependence on Resolution *r*

**Figure 13:**
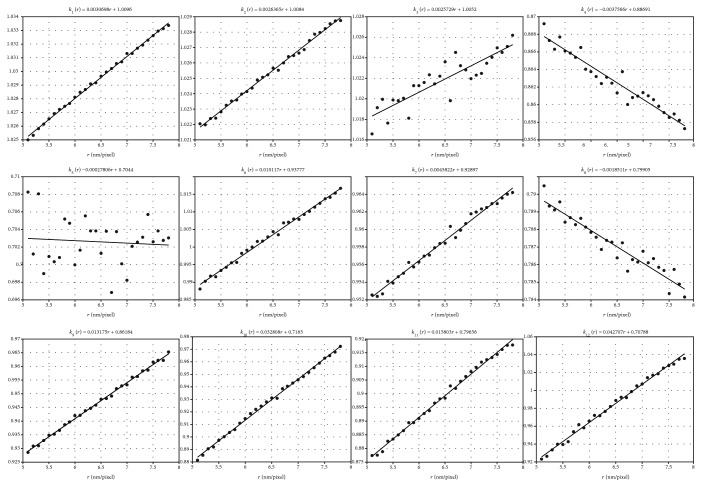
Linear fit of all *k*_1_ ~ *k*_12_ coefficients, with respect to resolution *r*, i.e., *k*_*j*_(*r*) = *mr* + *b*, as in equation (10).

With the convergence for all the *k*_*j*_ coefficients confirmed for all different *r*, the relationship for each *k*_*j*_(·) as a function of *r*, i.e., the linear fit for *k*_*j*_(*r*) results, is found in Figure 13.

It is clear that using the converged *k*_*j*_ values for a specified *r*, the following linear fit equation,

(10) $$\begin{array}{c} k\left(r\right)=mr+b, \end{array}$$

results with a table that contributes to all the twelve different coefficients; it is provided in Table 5.

### 3.3. Performance with Shape Modification Coefficient

The above sections result in the calibration correction in equation (10) and can be used for the unknown *l*_*c*_ estimation. In order to demonstrate such performance, the coefficient in equation (10) is used for the shape estimator *L*_S_ and compared alongside the DNA estimator *L*_DNA_ and the Freeman estimator *L*_F_.

All estimators *L*_S_, *L*_DNA_, and *L*_F_ are compared with different length *l*_*c*_ and resolution *r*. All the estimators are applied for the same simulated pixel images and compared against the readily known *l*_*c*_ for error calculation.

Tables 6, 7, and 8 outline the calculation error for different *r* settings. It shows that the *L*_S_ estimator has an averaged relative error maxed at 0.07%, performing with an order of magnitude difference from the *L*_DNA_ estimator, and two orders of magnitude smaller than *L*_F_. The relative error translates to an absolute value of maximum 0.20 nm for the *r* = 5.1 nm/pixel, well below the resolution, making *L*_S_ ideal for *l*_*c*_ estimation.

Since the error is averaged amongst the 100,000 samples provided, its standard deviation (STD) in nm is also an indicator for quantitative analysis. The *L*_S_ estimator also has a smaller standard deviation compared to both *L*_DNA_ and *L*_F_, against a growing *l*_*c*_ contour estimated.

## 4. Conclusion and Future Direction

This research provided a novel way to estimate digitized contour length, in a general way that is applicable towards all kinds of contour curvature. Utilizing a localized shape connection approach, and correct upon the local connectivity between pixels, this algorithm accounts for both resolution and the sample stiffness.

This research is general in the local 4-pixel segment identification method and extensible towards extension to more pixel elements. The general idea stands that a single-width pixel contour's digital shape recognition is applicable towards all images acquired from different systems, not only with the AFM family but also optical microscopy systems, electron microscopy systems, and many others.

Experimental verification is also needed for future research, provided with calibrated accurate sample length *l*_*c*_ from DNA samples or other biopolymer samples imaged with AFM systems.

## Figures and Tables

**Algorithm 1:**
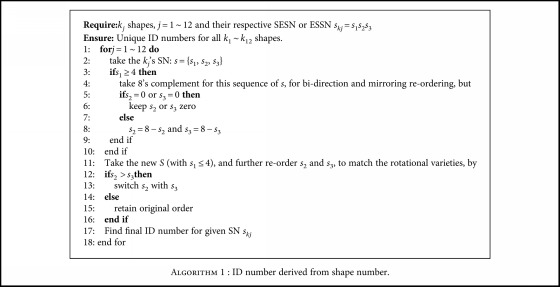
Algorithm 1 : ID number derived from shape number.

**Table 1:** Comparison of digital contour length methodology difference with biopolymer samples.

Author	Year	Estimator	Algorithm	Specific to DNA
Spisz et al. [8]	1998	Freeman estimator *L*_F_	*L*_F_ = *r*(*n*_e_+√2*n*_o_)	
Rivetti and Codeluppi [9]	2001	Kupla *L*_K_ and corner *L*_C_ estimator	*L*_K_ = *r*(0.948*n*_e_ + 1.343*n*_o_), *L*_C_ = *r*(0.980*n*_e_ + 1.406*n*_o_ − 0.091*n*_*c*_)	✓
Sanchez-Sevilla et al. [10]	2002	Curvature filtering	Coordinate transform to complex plane with Fourier transform	
Ficarra et al. [11]	2005	Pixel coordinate	Relocation of pixel representative coordinate *X*	
Rivetti [12]	2009	DNA estimator *L*_DNA_	*L*_DNA_ = *rC*_*f*_(*n*_e_+√2*n*_o_), *C*_*f*_ = 〈*l*_*c*_〉/*L*_F_	✓
Sundstorm et al. [13]	2012	Machine learning	*L*_ML_ = ∑{*n*_horz_, *n*_vert_, *n*_diag_, *n*_perp_, *n*_htcv_, *n*_tkcv_}	✓

**Table 2:** Example ID number retrieval for *k*_8_ shape numbers.

	Original *k*_8_ SN	116	772	727	161
Step one	Check *s*_1_, take 8's complement if *s*_1_ > 4	116	$\underset{8\text{'}s\text{ comp}}{\underset{⎵}{116}}$	$\underset{8\text{'}s\text{ comp}}{\underset{⎵}{161}}$	161
Step two	Reorder *s*_2_ and *s*_3_ digits when *s*_2_ > *s*_3_	116	116	1 $\underset{\text{reorder}}{\underset{⎵}{16}}$	1 $\underset{\text{reorder}}{\underset{⎵}{16}}$
	Final unique *k*_8_ ID			**116**	

**Table 3:** ID number derivation from SN for all *k*_1_ ~ *k*_12_ shapes.

Shape	*k*_1_	*k*_2_	*k*_3_	*k*_4_	*k*_5_	*k*_6_	*k*_7_	*k*_8_	*k*_9_	*k*_10_	*k*_11_	*k*_12_
ID number	**000even**	**000odd**	**017even**	**017odd**	**026**	**107even**	**107odd**	**116**	**206**	**277even**	**277odd**	**367**
Original SN	000	000	017 071	017 071	026 062	107 170	107 170	116 161	206 260	277 611	277 611	367 376
						701	701	727	602			512
						710	710	772	620			521

**Table 4:** Connection length for *l*_*k*_1__ ~ *l*_*k*_12__.

Shape	*k*_1_	*k*_2_	*k*_3_	*k*_4_	*k*_5_	*k*_6_
Length	1	√2	1	√2	√2	1
Shape	*k*_7_	*k*_8_	*k*_9_	*k*_10_	*k*_11_	*k*_12_
Length	√2	√2	√2	1	√2	√2

**Table 5:** Coefficient table of *m* and *b* in *k*_*j*_(*r*) = *mr* + *b*.

*k*(*r*)	*m*	*b*
*k*_1_	0.0030698	1.0096
*k*_2_	0.0026365	1.0084
*k*_3_	0.0025729	1.0052
*k*_4_	-0.0037566	0.88691
*k*_5_	-0.00027806	0.7044
*k*_6_	0.010117	0.93777
*k*_7_	0.0045822	0.92897
*k*_8_	-0.0018511	0.79905
*k*_9_	0.013175	0.86184
*k*_10_	0.032808	0.7165
*k*_11_	0.015803	0.79656
*k*_12_	0.042707	0.70788

**Table 6:** Error analysis of *L*_S_, *L*_DNA_, and *L*_F_, at *r* = 5.1 nm/pixel.

*r* = 5.1 nm/pixel	Error			
Estimator	*l*_*c*_(*nm*)	Relative (%)	Absolute (nm)	std (nm)
*L*_S_	340	0.05	0.19	3.6
	680	0.00001	0.00009	4.51
	1020	0.02	0.20	5.23

*L*_DNA_	340	0.26	0.99	4.71
	680	0.24	1.67	6.18
	1020	0.24	2.45	7.36

*L*_F_	340	3.42	11.63	4.86
	680	3.40	23.12	6.38
	1020	3.39	34.63	7.60

**Table 7:** Error analysis of *L*_S_, *L*_DNA_, and *L*_F_, at *r* = 6.4 nm/pixel.

*r* = 6.4 nm/pixel	Error			
Estimator	*l*_*c*_(nm)	Relative (%)	Absolute (nm)	std (nm)
*L*_S_	340	0.06	0.23	4.77
	680	0.0005	0.003	5.92
	1020	0.02	0.24	6.92

*L*_DNA_	340	0.35	1.21	5.80
	680	0.33	2.28	7.57
	1020	0.32	3.31	9.06

*L*_F_	340	2.87	9.78	5.94
	680	2.85	19.43	7.76
	1020	2.84	29.03	9.29

**Table 8:** Error analysis of *L*_S_, *L*_DNA_, and *L*_F_, at *r* = 7.7 nm/pixel.

*r* = 7.7 nm/pixel	Error			
Estimator	*l*_*c*_ (nm)	Relative (%)	Absolute (nm)	std (nm)
*L*_S_	340	0.07	0.24	5.93
	680	0.004	0.03	7.49
	1020	0.02	0.29	8.76

*L*_DNA_	340	0.43	1.47	6.93
	680	0.40	2.73	9.11
	1020	0.39	4.01	10.86

*L*_F_	340	2.32	7.91	7.06
	680	2.29	15.61	9.28
	1020	2.28	23.33	11.07

## Data Availability

The data used to support the findings of this study are included within the article.
